# Right Infectious Cavernous Sinus Thrombosis Complicated by Multiple Cerebrovascular Events in the Right Hemisphere

**DOI:** 10.7759/cureus.89505

**Published:** 2025-08-06

**Authors:** Kaito Kubota, Taiki Matsubayashi, Yuki Aizawa, Misako Furuki, Masato Obayashi

**Affiliations:** 1 Department of Neurology, National Hospital Organization Disaster Medical Center, Tokyo, JPN; 2 Department of Neurosurgery, National Hospital Organization Disaster Medical Center, Tokyo, JPN

**Keywords:** bacterial meningitis, cavernous sinus thrombosis, cerebral infarction, sphenoid sinusitis, streptococcus intermedius, subarachnoid hemorrhage

## Abstract

Bacterial meningitis and infectious cavernous sinus thrombosis (CST) are both life-threatening central nervous system infections, often caused by sinusitis. While cerebrovascular complications are well-recognized in bacterial meningitis, their association with CST is rare.

A 69-year-old man presented with a 19-day history of headache, followed by diplopia. On the day of admission, he developed left hemiparesis. On examination, he had mild right eye abduction impairment, dysarthria, mild left hemiparesis with left facial paralysis, and nuchal rigidity. At admission, brain computed tomography revealed sinusitis and a subarachnoid hemorrhage (SAH) in the right Sylvian fissure, while catheter angiography demonstrated right CST without cerebral aneurysm. Subsequent brain magnetic resonance imaging (MRI) revealed cerebral infarction in the right lateral striate artery territory. Cerebrospinal fluid (CSF) analysis showed elevated neutrophils, increased protein, and a decreased CSF/serum glucose ratio. The patient was treated with antimicrobial agents and heparin. Endoscopic sinus surgery was performed on day five after admission. *Streptococcus intermedius* (*S. intermedius*) was identified from sinus pus and blood cultures, leading to a diagnosis of bacterial meningitis. On day nine, a follow-up MRI was performed due to progressive left-sided weakness since day three. Contrast-enhanced imaging showed resolution of the cavernous sinus thrombus but revealed an extensive acute ischemic stroke in the right middle cerebral artery region. Consequently, heparin was discontinued, and cilostazol was initiated. Although his headache and diplopia improved, left-sided hemiplegia persisted. He was transferred to a rehabilitation hospital on day 32 after admission.

In this case, the patient presented with right CST and bacterial meningitis, accompanied by SAH and acute ischemic stroke in the right cerebral hemisphere. CST and bacterial meningitis were likely secondary to sinusitis. The initial right temporal headache was attributed to inflammatory involvement of the internal carotid artery due to CST, which also contributed to cerebrovascular complications. The detected *S. intermedius*, a highly invasive pathogen, may have played a role in the extensive vascular complications. Though rare, CST requires careful monitoring for ipsilateral cerebrovascular complications.

## Introduction

Cavernous sinus thrombosis (CST) is a rare but life-threatening condition, accounting for 1-4% of cerebral venous thromboses, with an annual incidence of approximately two to 13 cases per million people [1]. It is most commonly caused by an infection, with the direct inflammatory spread from ethmoid or sphenoid sinusitis responsible for 50% of CST cases [1]. The clinical presentation of CST varies widely, but common symptoms include fever, headache, ocular manifestations, and cranial nerve palsy [1].

Bacterial meningitis is another life-threatening central nervous system (CNS) infection, characterized by fever, headache, stiff neck, nausea, vomiting, and confusion. Notably, 34% of patients with bacterial meningitis have a history of sinusitis or otitis media [2], underscoring sinusitis as a significant risk factor for CNS infections.

CNS infections are frequently associated with stroke, including cerebral infarction, intracerebral hemorrhage, and subarachnoid hemorrhage (SAH). Indeed, cerebral infarction has been reported in up to 25% to 31% of bacterial meningitis cases [3]. However, reports of stroke occurring in conjunction with CST are limited. Here, we present a case of bacterial meningitis associated with infectious CST caused by *Streptococcus intermedius* (*S. intermedius*) secondary to sinusitis. The patient developed right-sided CST, along with acute ischemic stroke and SAH, both localized to the right cerebral hemisphere. This case provides valuable insights into the mechanisms underlying stroke occurrence in CNS infections.

## Case presentation

A 69-year-old man presented to our hospital with left-sided difficulty in exerting strength. He had experienced a headache in the right temporal region for 19 days before admission and had visited a local neurosurgery department on day four after symptom onset. Brain magnetic resonance imaging (MRI) at that time revealed sinusitis in the maxillary and ethmoidal sinuses, but no cerebrovascular lesions were detected. He was prescribed non-steroidal anti-inflammatory drugs. On day 11 after onset, he began experiencing double vision and consulted a local ophthalmologist, who found no abnormalities. On the day of admission, he noticed slurred speech upon waking and difficulty elevating the left corner of his mouth. That evening, while undressing, he experienced difficulty moving his left upper and lower limbs. Suspecting a stroke, he was transported to our hospital for emergency treatment. His medical history included hypertension, diabetes mellitus, and prostate cancer, which had been treated with radiation therapy and remained stable.

On arrival, his vital signs were as follows: body temperature, 36.6°C; blood pressure, 135/68 mmHg; heart rate, 90 beats/min; respiratory rate, 12 breaths/min; and oxygen saturation, 96% on room air. He was conscious and alert, with a Glasgow Coma Scale score of E4V5M6. Neurological examination revealed bilaterally round and equal pupils with brisk light reflexes. Mild abduction impairment (-2) of the right eye due to abducens nerve palsy was noted. Additionally, he exhibited mild left hemiparesis with facial paralysis (a Manual Muscle Testing score of 4) and dysarthria caused by facial paralysis [4]. No other cranial nerve deficits were observed. A positive Babinski sign was observed on the left side. Nuchal rigidity was present, but other meningeal signs, such as Kernig's sign and Brudzinski's sign, were not observed. No sensory impairment in the extremities or bladder and rectal dysfunctions were observed.

Blood tests revealed an elevated white blood cell count (WBC) of 22,300/μL, with 91.2% neutrophils, and a C-reactive protein level of 13.80 mg/dL. D-dimer was also elevated at 6.6 mg/dL. Blood glucose was high at 254 mg/dL, with an increased glycated hemoglobin level of 7.5%.

Cerebrospinal fluid (CSF) analysis revealed a mildly yellow appearance with a normal opening pressure of 180 mmH₂O. The CSF showed an elevated WBC of 2,008/3μL, predominantly polynuclear cells (1,552/3μL), an increased protein level of 141.3 mg/dL, and a low CSF/serum glucose ratio of 0.37. The initial laboratory findings are summarized in Table 1.

**Table 1:** Laboratory parameters analyzed in the serum and CSF. CSF: cerebrospinal fluid, *S. intermedius*: *Streptococcus intermedius*.

	Laboratory parameters	Value (units)	Reference value
Serum	White blood cell	22,300/μL	4,000-10,000
	Neutrophils	91.2%	40-70
	Fibrinogen	660 mg/dL	150-400
	D-dimer	6.6 μg/mL	<1.0
	C-reactive protein	13.80 mg/dL	<0.5
	Blood urea nitrogen	20.0 mg/dL	8-20
	Creatinine	0.59 mg/dL	0.65-1.07
	Glucose	254 mg/dL	73-109
	Glycated hemoglobin	7.5%	4.9-6.0
	Interferon-gamma release assay	Negative	Negative
	β-d-glucan	6.7 pg/mL	<20
	Candida antigen	< 0.02 U/mL	< 0.05
	Aspergillus antigen	0.1	<0.5
	Blood bacterial culture	S. intermedius	Negative
	Blood fungal culture	Negative	Negative
CSF	Color	Mildly yellow	-
	CSF pressure	180 mmH20	70-180
	White blood cell	2,008/3μL	0-15
	Polymorphonuclear leukocyte	1,552/3μL	-
	Mononuclear leukocyte	456/3μL	-
	Protein	141.3 mg/dL	15-45
	CSF/serum glucose ratio	0.37	0.6-0.8
	Bacterial culture	Negative	Negative
	Fungal culture	Negative	Negative
Sinus pus	Bacterial culture	S. intermedius	Negative
	Fungal culture	Negative	Negative
Sinus mucosa	Cytology	No malignancy	-

**Figure 1:**
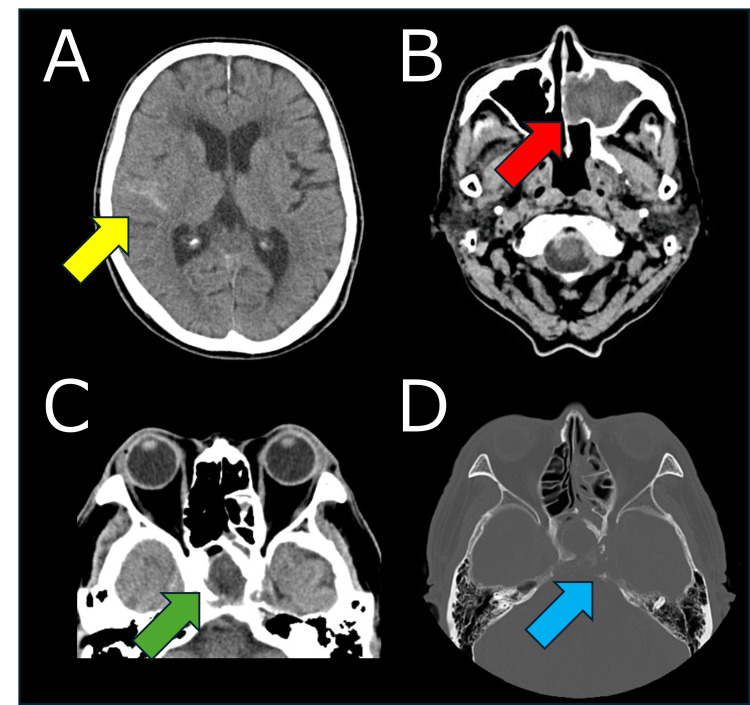
Brain CT at Admission. The initial CT revealed a hyperdense area in the right Sylvian fissure (A; yellow arrow) and a soft tissue shadow in the left maxillary (B; red arrow) and sphenoid (C; green arrow) sinuses, partially accompanied by osteolytic changes (D; blue arrow). CT: computed tomography.

Brain computed tomography (CT) at admission showed a hyperdense area in the right Sylvian fissure, suggestive of SAH (Figure 1A). Additionally, soft tissue opacities were detected in the left maxillary sinus (Figure 1B) and ethmoidal sinus (Figure 1C), accompanied by osteolytic changes in the bone structures bordering the sphenoid sinus (Figure 1D).

**Figure 2:**
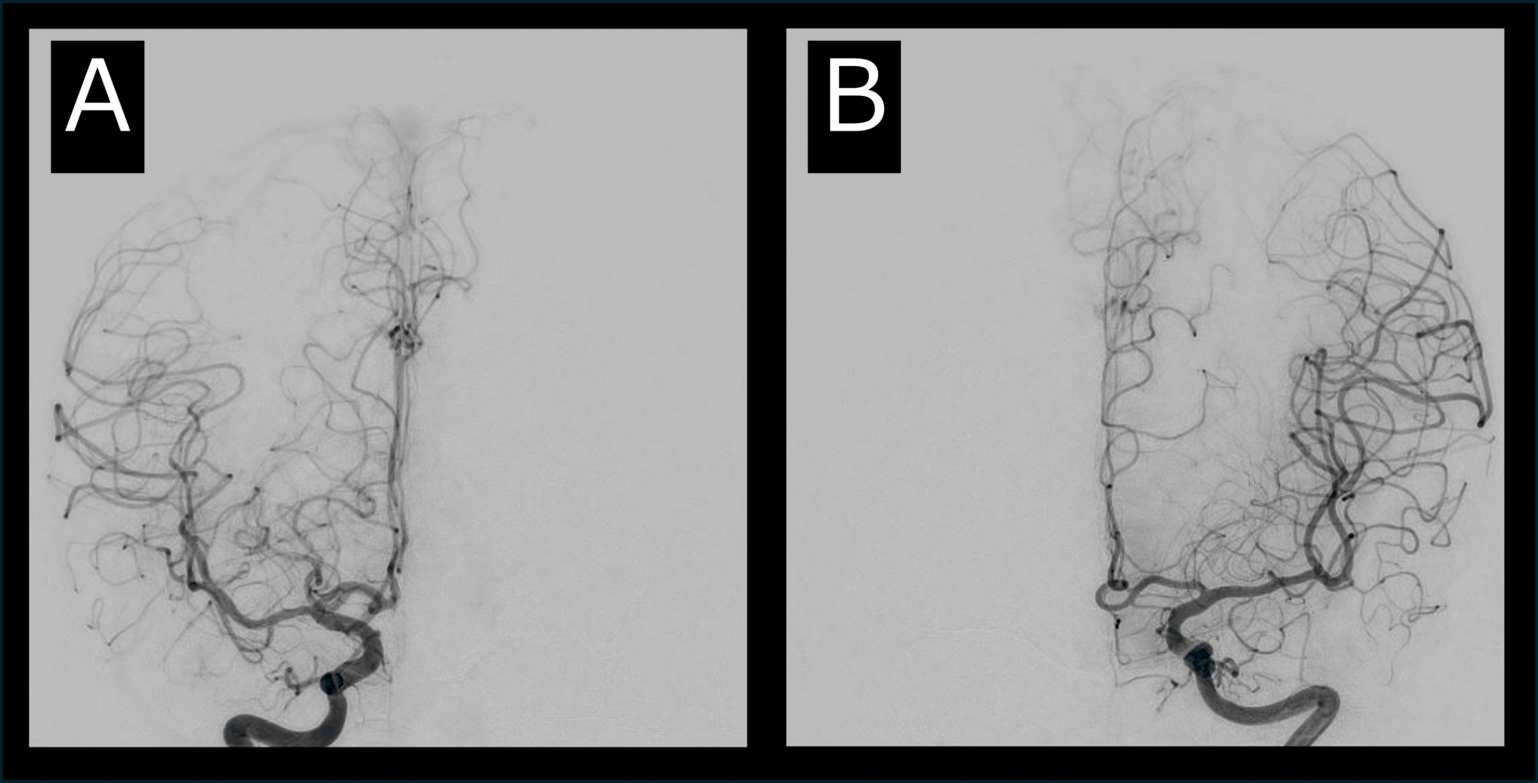
Catheter Angiography in the Arterial Phase at Admission. Catheter angiography in the arterial phase revealed no aneurysm or arterial occlusion from either the right (A) or left (B) internal carotid artery.

Catheter angiography was performed for the evaluation of SAH, but no cerebral aneurysm was detected in the arterial phase (Figure 2).

**Figure 3:**
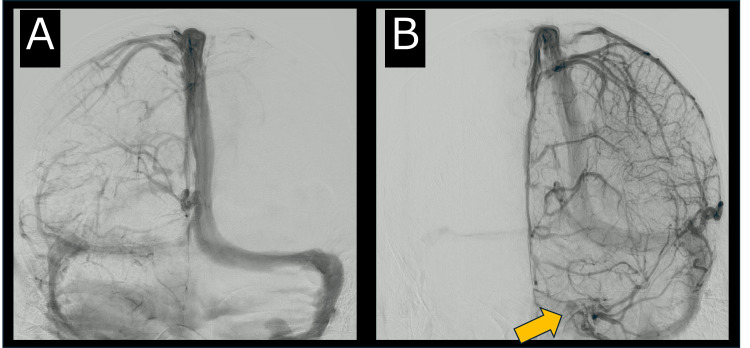
Catheter Angiography in the Venous Phase at Admission. Catheter angiography in the venous phase revealed an absence of venous return from the right superficial middle cerebral vein to the right cavernous sinus (A), whereas venous return from the left superficial middle cerebral vein to the left cavernous sinus was normal (B; orange arrow).

However, the venous phase revealed a lack of venous return from the right superficial middle cerebral vein to the right cavernous sinus, leading to a diagnosis of right CST (Figure 3).

**Figure 4:**
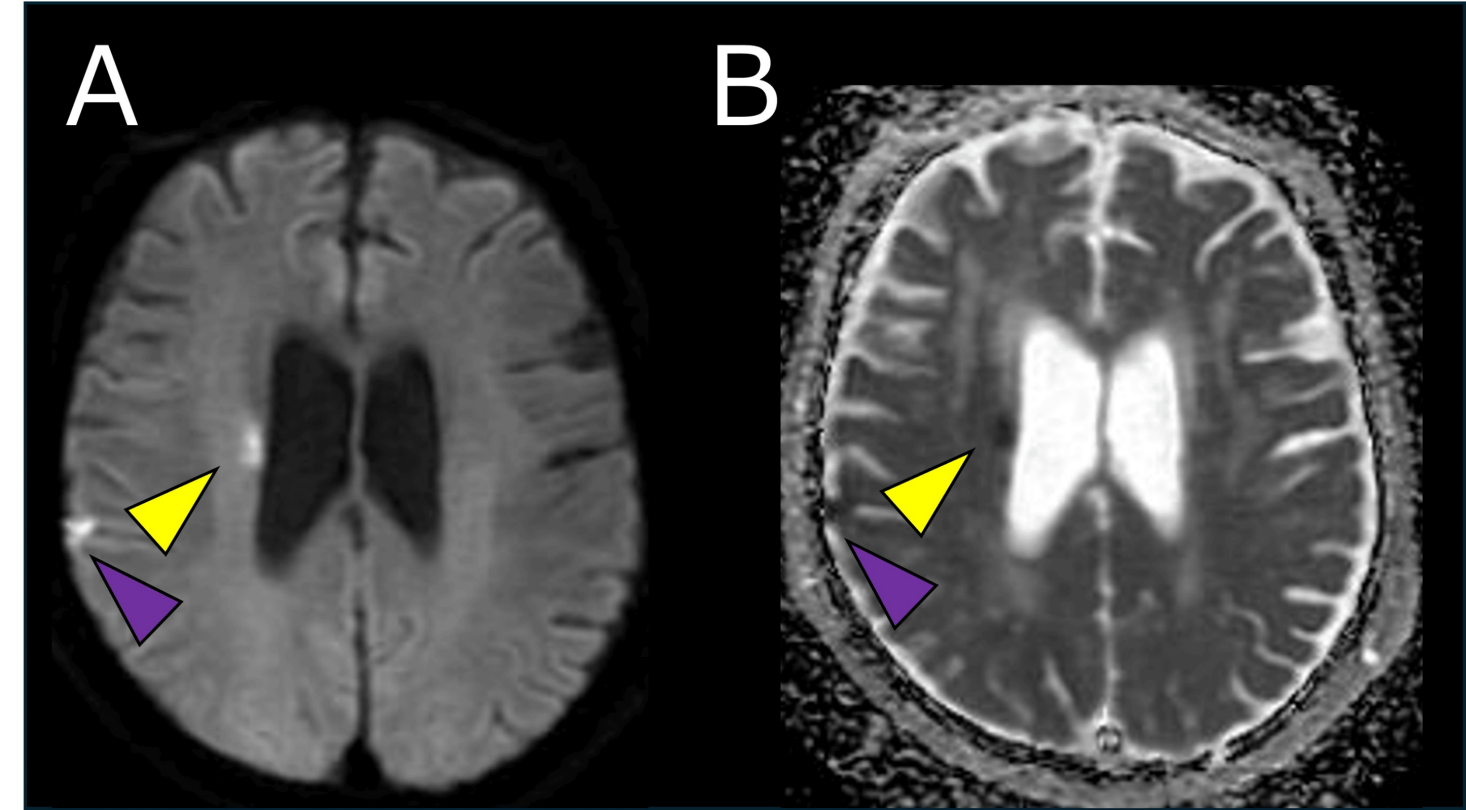
Brain MRI at Admission. Diffusion-weighted imaging (A) revealed high signal intensity in the right lateral striate artery territory, with corresponding low values on apparent diffusion coefficient mapping (B) (yellow arrowheads). The high signal observed in the right cerebral cortex on diffusion-weighted imaging (A) and the corresponding low value on the apparent diffusion coefficient map (B) appeared hypointense on the T2-weighted image (not shown), suggesting hemorrhage (purple arrowheads). MRI: magnetic resonance imaging.

Non-contrast MRI at admission showed high signal intensity on diffusion-weighted imaging (DWI) and low values on apparent diffusion coefficient (ADC) mapping in the right lateral striate artery territory, consistent with acute ischemic stroke (Figure 4). However, magnetic resonance angiography (MRA) revealed no major arterial stenosis or occlusion.

**Figure 5:**
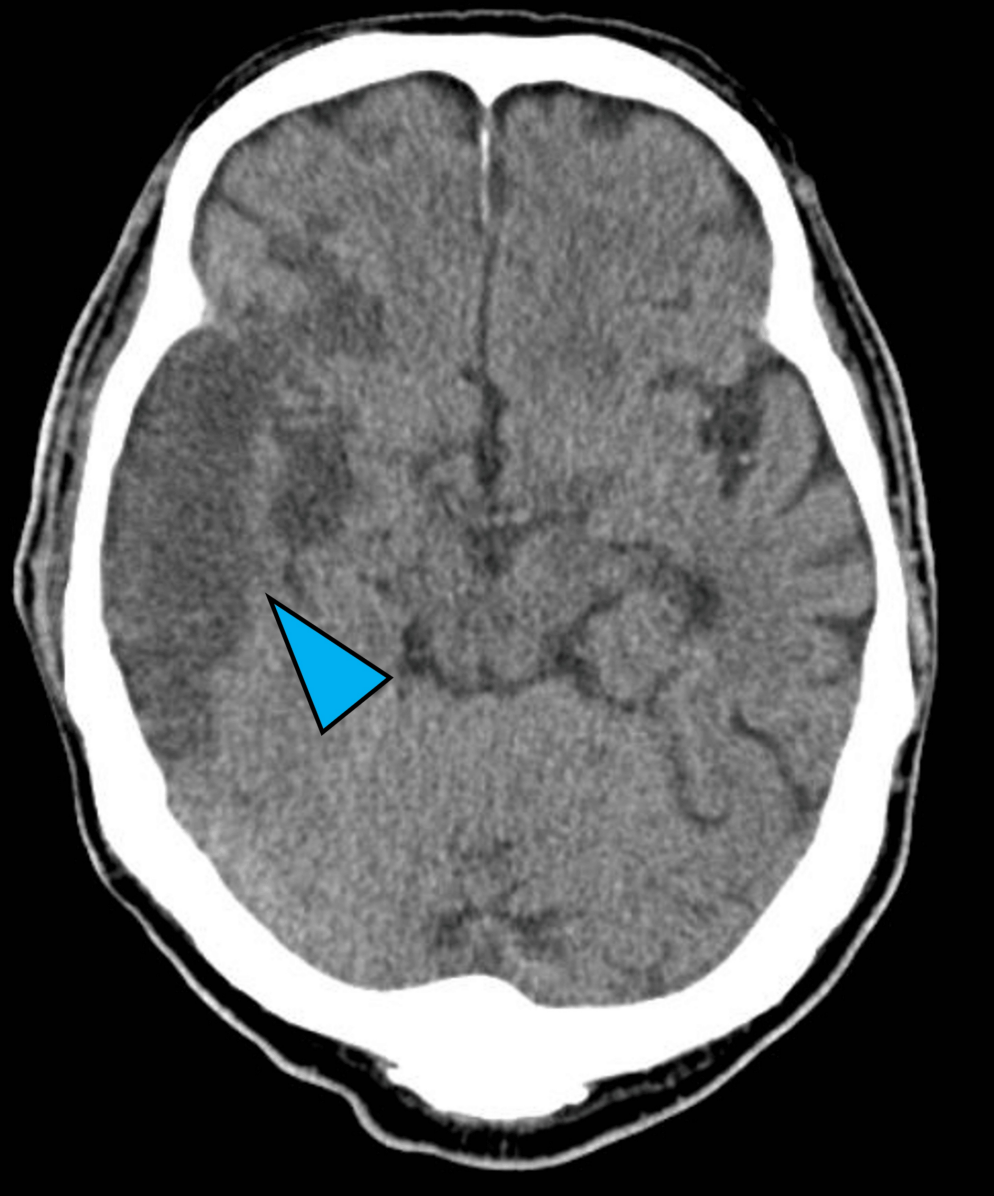
Brain CT on Day 6. Follow-up CT revealed extensive low-density areas in the right MCA region (blue arrowhead). CT: computed tomography, MCA: middle cerebral artery.

Based on these findings, the patient was diagnosed with bacterial meningitis and infectious CST associated with SAH and cerebral infarction. Empirical antibacterial therapy with ceftriaxone, vancomycin, and ampicillin was initiated for bacterial meningitis, along with anticoagulation therapy using unfractionated heparin for CST. Dexamethasone was administered short-term until day two. On day two after admission, the patient exhibited a decreased level of consciousness (Glasgow Coma Scale: E3V3M6). Given the CT findings with osteolytic changes in the bone structures bordering the sphenoid sinus, a fungal infection could not be ruled out, so antifungal therapy with voriconazole and amphotericin B was additionally initiated. While his headache and diplopia gradually improved, his left-sided muscle strength declined from a Manual Muscle Test grade of 4 to 1 by day three. On day five, endoscopic sinus surgery was performed to drain the sphenoid and maxillary sinuses. On day six, brain CT revealed extensive low-density areas in the right middle cerebral artery (MCA) region, suggesting an enlarged ischemic stroke (Figure 5). In contrast, the SAH showed signs of improvement. Examination by the department of oral and maxillofacial surgery revealed apical periodontitis of the upper left tooth, which was considered a possible cause of the left-sided sinusitis.

**Figure 6:**
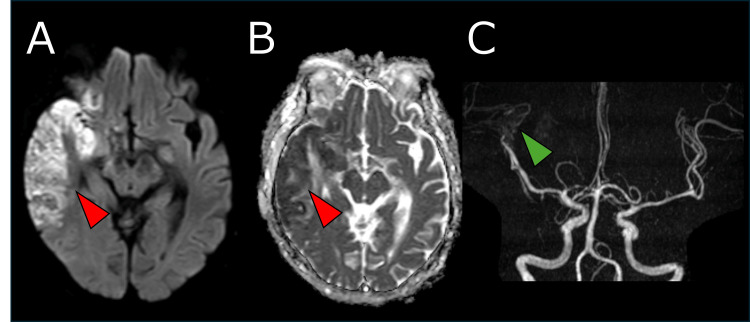
Brain MRI on Day 9. Follow-up diffusion-weighted imaging (A) showed extensive high signal intensity in a widespread area of the right MCA territory, with corresponding low values on apparent diffusion coefficient mapping (B) (red arrowheads). MRA demonstrated poor visualization of the M2 segment of the MCA (C; green arrowhead). MCA: middle cerebral artery, MRA: magnetic resonance angiography, MRI: magnetic resonance imaging.

By day nine, the brain MRI showed extensive high signal intensity on DWI-MRI with low ADC values in the right MCA region, consistent with CT findings (Figure 6A, 6B). MRA demonstrated poor visualization of the M2 segment of the MCA (Figure 6C). Gadolinium-enhanced T1-weighted MRI showed no significant contrast defects or asymmetry in the cavernous sinus, indicating CST resolution.

Consequently, heparin was discontinued, and cilostazol was initiated for ischemic stroke management, but was switched to clopidogrel due to headache as a side effect. *S. intermedius*, which was sensitive to various antimicrobial agents, was cultured from blood and sinus pus. Fungal cultures from blood, CSF, and sinus pus were all negative. Additionally, no malignancy was detected in the sinus mucosa. Based on these results, vancomycin and amphotericin B were discontinued on day five, ceftriaxone on day nine, and voriconazole on day 10, while ampicillin was continued until day 16. By day 17, the patient’s headache had completely resolved, and his level of consciousness had fully cleared. However, as left-sided hemiplegia persisted, he was transferred to another hospital on day 32 after admission for continued rehabilitation. He remained alive one year after onset, although left hemiparesis persisted. Details of the treatment course are shown in Table 2.

**Table 2:** Summary of treatment.

Treatment	Dose	Duration
Antibiotics		
Vancomycin	(day 1 – day 3) 1g q12h (day 4 – day 5) 0.75g q12h	day 1 – day 5
Amphotericin B	150mg q24h	day 2 – day 5
Ceftriaxone	2g q12h	day 1 – day 9
Voriconazole	200mg q12h	day 2 – day 10
Ampicillin	2g q4h	day 1 – day 16
Dexamethasone	9.9mg q6h	day 1 – day 2
Antithrombotic drug		
Unfractionated heparin	10,000 IU/day	day 1 – day 9
Cilostazol	200 mg/day	day 10 – day 25
Clopidogrel	75 mg/day	day 26 –
Endoscopic sinus surgery	-	day 5

## Discussion

In the present case, the patient developed right-sided CST, followed by various cerebrovascular complications, including SAH and cerebral infarction, in the right cerebral hemisphere. To our best knowledge, a total of seven adult cases of CST complicated by cerebrovascular events with accessible clinical details have been reported between 2006 and 2025 [5-11]. A summary of these cases is presented in Table 3. Including our case, five involved unilateral CST, and all exhibited cerebrovascular lesions on the same side as the thrombosis. Cerebrovascular complications of CST are diverse and may include cerebral infarction, intracerebral hemorrhage, and SAH. While pediatric CST cases are also prone to such complications, they are generally associated with more favorable outcomes [12]. In contrast, adult cases tend to have a poorer prognosis. Treatment typically includes anticoagulants or antithrombotic agents, antibiotics, and surgical drainage when sinusitis is the underlying cause. In our case, timely intervention, including endoscopic sinus surgery, may have contributed to the favorable clinical course. Anticoagulation therapy with heparin is generally not used in cases of SAH. However, in this case, the absence of an aneurysm suggested that the SAH was likely secondary to inflammation extending from CST. Given this pathophysiological context, we prioritized treatment of the underlying cause and initiated heparin therapy to address the CST. The extent of the SAH was limited, and careful radiological follow-up was conducted to monitor for any progression. Fortunately, no worsening of the hemorrhage was observed throughout the clinical course. Notably, a previous case report has documented the use of urokinase for CST even in the presence of SAH and intracerebral hemorrhage [6]. However, standard treatment protocols for CST with cerebrovascular complications have not yet been established, and further accumulation of case reports is warranted.

**Table 3:** Cases of cerebrovascular disease secondary to CST. ACA: anterior cerebral artery, CST: Cavernous sinus thrombosis, CNS: central nervous system, F: female, ICA: internal carotid artery, LSA: lateral striatal artery, M: male, MCA: middle cerebral artery, MRSA: Methicillin-resistant Staphylococcus aureus. PCA: posterior cerebral artery, SAH: subarachnoid hemorrhage, *S. constellatus*: *Streptococcus constellatus*, *S. intermedius*: *Streptococcus intermedius*.

Study	Year	Age	Sex	Neurological examination	CST side	Infectious source	CNS complications	Causative bacteria	Treatment	Outcome
Matsuo R et al. [5]	2006	65	F	Headache, left hemiparesis	Right	Sphenoid and ethmoid sinusitis without description of side	Right subdural empyema, hemorrhagic infarction in the right frontotemporal lobe	-	Antibiotics, drainage of the sphenoid sinus	Died
Kurosu A et al. [6]	2007	37	M	Left hemiplegia, dysarthria, headache	Left	Non-infectious	SAH, left putaminal hemorrhage	-	Urokinase, hematoma removal	Remaining severe disability after one year
Kotagiri R et al. [7]	2020	35	F	Neck stiffness, headaches, right cranial nerve palsy (VI), left cranial nerve palsy (III)	Bilateral	Unknown	Left PCA infarction	MRSA	Antibiotics, heparin	Brain death
Young KS et al. [8]	2021	61	F	Right hemiplegia, aphasia	Left	Left sphenoid sinusitis	Left LSA infarction, left superior ophthalmic vein thrombosis	S. constellatus	Antibiotics, heparin	Improved aphasia, requiring a cane for walking
Rosa F et al. [9]	2022	60	M	Bilateral abduction limitation (VI cranial nerve palsy)	Bilateral	Bilateral orbital cellulitis	Intraparenchymal hemorrhage in the right temporal lobe, brainstem venous infarction	MRSA	Antibiotics, anticoagulants	Died
He P et al. [10]	2023	46	M	Decreased right vision, headache	Right	Right sphenoid sinusitis	Right ICA infarction, SAH with right ACA aneurysm	No description	Antibiotics, antiplatelet drugs, aneurysm clipping	Died two months after onset
Li CR et al. [11]	2024	70	M	Right eye movement disorder	Bilateral	Sphenoid sinusitis without description of side	SAH, left transverse sigmoid sinus thrombosis, bacterial meningitis	No description	Antibiotics	Died five days after admission
Our case		69	M	Left hemiplegia, right abduction limitation (VI cranial nerve palsy)	Right	Left sphenoid sinusitis	Right LSA and MCA infarction, SAH at right cerebral hemisphere, bacterial meningitis	S. intermedius	Antibiotics, heparin, antiplatelet drugs, endoscopic sinus surgery	Improved abduction limitation, persistent left hemiplegia

The cavernous sinus is adjacent to the sphenoid sinus and contains the internal carotid artery along with multiple cranial nerves, including the oculomotor, trochlear, ophthalmic, maxillary, and abducens nerves. Cerebrovascular disorders associated with CST are thought to arise from inflammatory spillover through the internal carotid artery or stenosis caused by inflammation [13]. In this case, right-sided head pain was the initial symptom, and MRI performed four days after onset showed no abnormalities. Given that the detection rate of SAH on fluid-attenuated inversion recovery MRI within five days of onset is reported to be 100% [14], SAH was ruled out as the cause of the headache. Additionally, sinusitis was considered an unlikely cause due to its location contralateral to the headache. Similarly, bacterial meningitis was deemed improbable, as it typically presents with diffuse rather than unilateral headache. Therefore, inflammatory spillover to the internal carotid artery and its branches due to the right cavernous sinus infection likely caused the headache. Furthermore, the onset of diplopia, likely due to abducens nerve palsy, eight days before admission suggests that CST developed prior to the cerebrovascular events. In conclusion, sinusitis led to the development of right infectious CST, which subsequently triggered headache via inflammation of the internal carotid artery, followed by abducens nerve palsy. The ongoing inflammation of the internal carotid artery likely extended to its branches, resulting in acute cerebral infarction in the lateral striate artery territory and SAH in the Sylvian fissure. Bacterial meningitis likely developed secondary to infectious CST and sinusitis with bone destruction. SAH can induce cerebral infarction through vasospasm of major arteries, and the extensive MCA infarction observed during hospitalization may have been a secondary effect of SAH. However, it is unlikely that cortical SAH alone would selectively cause vasospasm in the penetrating artery territory. Instead, both SAH and infarction in the lateral striate artery territory are more plausibly explained by inflammation spreading through the internal carotid artery.

Interestingly, in this case, sphenoid sinusitis and CST developed contralaterally. One possible explanation is a left-right asymmetry in sphenoid sinus size. Sphenoid sinus pneumatization is often asymmetrical, with reported variations in size and shape between the left and right sides [15]. In a previous case of left CST associated with right sphenoid sinusitis, the right sphenoid sinus was predominant and adjacent to the left cavernous sinus [16]. Similarly, in our case, brain CT confirmed that the left sphenoid sinus was dominant and adjacent to the right cavernous sinus, plausibly explaining the contralateral occurrence.

The sensitivity of CSF culture in bacterial meningitis is not 100% [17], and cases of central nervous system infections caused by *S. intermedius *with negative CSF cultures have been reported [18,19]. In our case, *S. intermedius* was identified in both blood cultures and pus cultures obtained from the paranasal sinus, which showed evidence of bone destruction. Considering these findings, along with the patient's clinical response to antimicrobial therapy, we diagnosed bacterial meningitis caused by *S. intermedius*.

*S. intermedius*, the causative organism in this case, is a common intraoral bacterium capable of producing a cytotoxin specific to humans and often leading to abscess formation [20]. Risk factors for its invasive infections include male sex, aging, diabetes, liver cirrhosis, and malignancy [21]. In this case, the patient had a history of diabetes and prostate cancer, which may have contributed to the development of *S. intermedius* infection. While bacterial meningitis caused by *S. intermedius* is rare, similar invasive cases have been reported [18]. While the high invasiveness of *S. intermedius*, the causative pathogen in this case, might have contributed to the complex CST complications, the potential role of *S. intermedius* underlying cerebrovascular complications in CST needs to be further evaluated.

## Conclusions

We experienced a case of right CST complicated by bacterial meningitis, which led to right hemisphere SAH and acute ischemic stroke. Although adult cases of CST complicated by cerebrovascular events are often associated with poor prognosis, the timely combination of anticoagulant therapy, antimicrobial treatment, and surgical intervention in this case may have contributed to a favorable outcome. The mechanism for the cerebral vascular diseases associated with ipsilateral CST in the present case was thought to be inflammation through the internal carotid artery. Although the sinusitis was located on the contralateral side of CST, anatomical differences in the size of the sphenoid sinus may explain this. The high invasiveness of *S. intermedius*, the causative pathogen in this case, might have contributed to the complex CST complications. Despite its rarity, ipsilateral cerebrovascular complications should be considered when diagnosing CST.
